# Non-Pharmacological Treatments in Paediatric Migraine

**DOI:** 10.3390/jcm13051278

**Published:** 2024-02-23

**Authors:** Valentina Baglioni, Fabiola Bozza, Annachiara Beatrice, Noemi Cameli, Elisa Maria Colacino Cinnante, Giuliana Lentini, Noemi Faedda, Giulia Natalucci, Vincenzo Guidetti

**Affiliations:** Child Neurology and Psychiatry Unit, Department of Human Neuroscience, Sapienza University, Via dei Sabelli 108, 00185 Rome, Italy; fabiola.bozza@uniroma1.it (F.B.); annachiara.beatrice@uniroma1.it (A.B.); noemi.cameli@uniroma1.it (N.C.); elisamaria.colacinocinnante@uniroma1.it (E.M.C.C.); giuliana.lentini@uniroma1.it (G.L.); noemi.faedda@uniroma1.it (N.F.); giulia.natalucci@uniroma1.it (G.N.); vincenzo.guidetti@uniroma1.it (V.G.)

**Keywords:** non-invasive neurostimulation, nutraceuticals, behavioural approaches, paediatric migraine

## Abstract

Psychological, social, and biological aspects contribute synergistically to the maintenance and chronicity of pain in primary headaches. An integrated intervention seems to be the most appropriate in the management of these conditions, taking advantage not only of pharmacological strategies, but also of different approaches according to the global assessment and patient necessities. In this perspective, non-pharmacological treatments are becoming increasingly used to overcome these issues also in paediatric migraine treatment. Particularly, nutraceuticals, non-invasive neuromodulation, and behavioural approaches are well tolerated and of potential interest. This paper aims to present the main approaches reported in the literature in the management of migraine in children and adolescents presenting an up-to-date review of the current literature. We therefore performed a narrative presentation for each of these three categories: nutraceuticals (riboflavin; magnesium; melatonin; vitamin D; coenzyme Q10; and polyunsaturated fatty acid); non-invasive neuromodulation (trigeminal nerve stimulator; non-invasive vagal nerve stimulation; transcranial magnetic stimulation; and remote electrical neuromodulation), and behavioural therapies (biofeedback; cognitive behavioural therapy; and mindfulness-based therapy). These approaches are increasingly seen as a valid treatment option in primary headache management also in paediatrics, avoiding medication overuse and drug treatment contraindications.

## 1. Introduction

Primary headaches and migraine are commonly reported in paediatric age, with an increasing trend from early childhood to adolescence; the prevalence of chronic daily headache indeed matches the one reported in adults, up to 2%.

**Figure 1:**
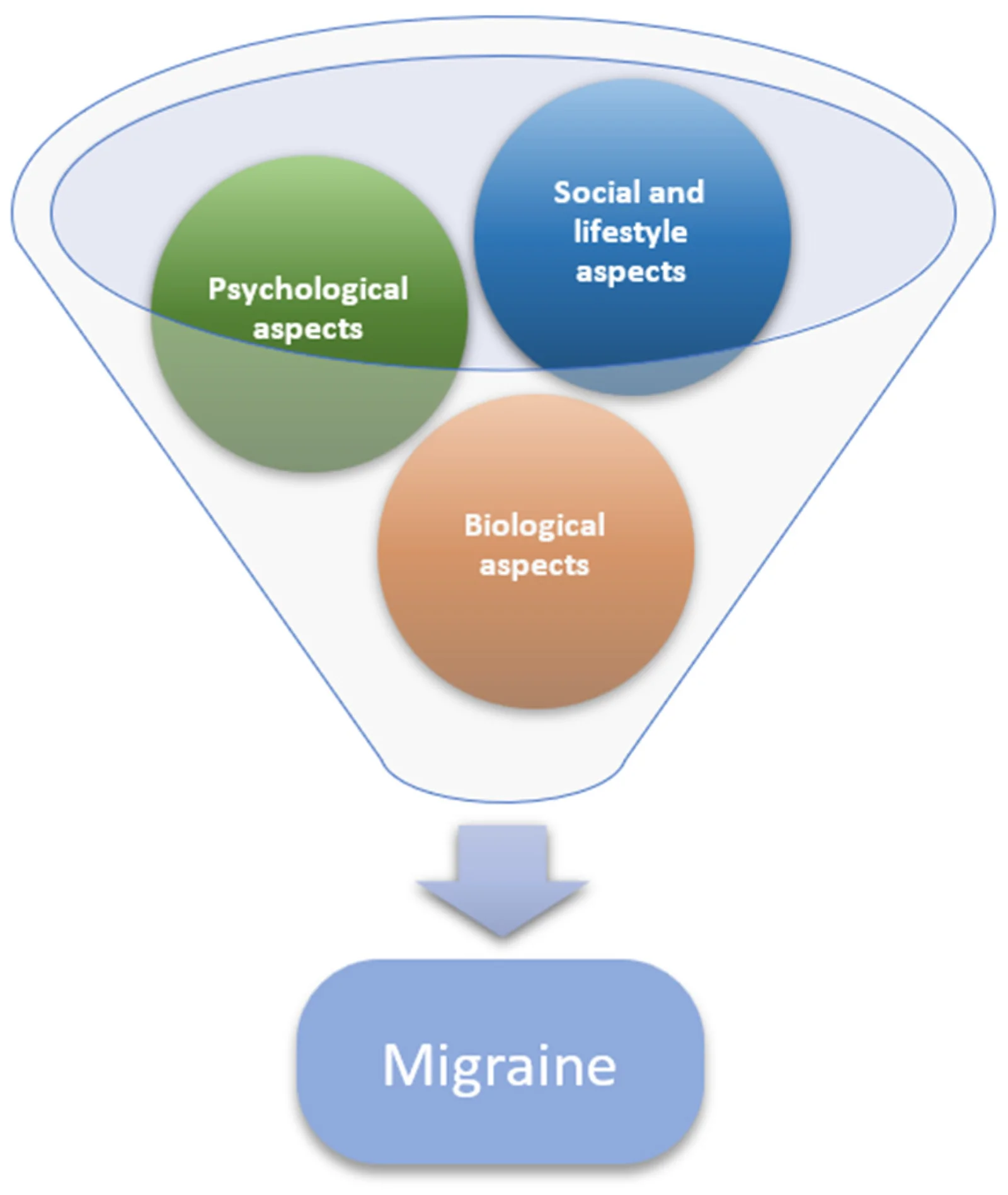
Migraine biopsychosocial model.

Psychological, social, and biological aspects contribute synergistically to the maintenance and chronicity of pain in primary headaches; in this perspective, an integrated intervention seems to be the most appropriate in the management of these conditions, taking advantage not only of pharmacological strategies but also of different approaches according to the global assessment and patient necessities (Figure 1).

Psychiatric comorbidities, epilepsy, and sleep disorders have a biological mechanism that shares similarities with migraines [1]. Furthermore, individuals who are predisposed to migraines and have comorbidities such as obesity and inflammatory diseases may experience an overt manifestation of migraine or a high migraine burden [1]. Psychiatric disorders that are linked to migraine include anxiety, depression, substance use disorder, bipolar disease, PTSD, and occasionally psychosis. Depression seems to be particularly linked to the chronicity of migraines among psychiatric conditions [1]. 

Based on the biopsychosocial model, an effective strategy for treating migraine should be targeted at the individual needs, considering overall the factors involved in the onset and persistence of migraine disorder. Thus, it is no longer appropriate to think of it only as an alternative or complementary treatment, but rather as one of the possible first-line treatment strategies for migraine, especially among children and adolescents. In this perspective, an integrated intervention seems to be the most appropriate in the management of these conditions, taking advantage not only of pharmacological strategies, but also of different approaches according to the global assessment and patient necessities (Figure 1).

Concerning non-pharmacological treatments of migraine and primary headaches, the updated paediatric guidelines published by the American Academy of Neurology show several limitations, not only due to the small number of homogeneous randomized clinical trials but also to the high efficiency of placebo strategies in children, with an efficacy up to almost 50% of cases, nearly matching the results of pharmacological therapies [2].

In adults, grade A evidence has been assigned to the supplementation of new non-pharmacological treatments for cognitive behavioural therapy, biofeedback, and relaxation therapy [3]. 

However, clinical studies on migraine non-pharmacological treatment in children [4,5,6,7,8] showed interesting initial results, both on primary endpoints as with a significant reduction in attack frequency and intensity and on secondary endpoints related to improvements in disability, quality of life, depression, and anxiety symptoms.

In this narrative review, we presented an up-to-date review of the literature about several non-pharmacological therapeutic approaches in the paediatric age (between 5 and 18 years old), such as nutraceuticals, lifestyle modification approaches, bio-behavioural treatments, educational interventions, psychotherapy, and non-invasive neuromodulation approaches (Table 1).

## 2. Materials and Methods

We reviewed the available literature on non-pharmacological approaches to paediatric migraine, performing a narrative presentation focused on each of these three categories: nutraceuticals (riboflavin; magnesium; melatonin; vitamin D; coenzyme Q10; and polyunsaturated fatty acid—PEA); non-invasive neuromodulation (trigeminal nerve stimulator—eTNS; non-invasive vagal nerve stimulation nVNS; transcranial magnetic stimulation—TMS; and remote electrical neuromodulation—REN), and behavioural therapies (biofeedback—BFB; cognitive behavioural therapy—CBT; mindfulness-based therapy—MDT). For each category, a narrative presentation will be made of the approaches published up to date, focusing both on the primary and secondary outcomes reported in the various studies.

## 3. Results

### 3.1. Lifestyle Modifications and Nutraceuticals

Evidence-based recommendations for migraine prevention in children include lifestyle assessment. Several behavioural factors, including sleep hygiene, diet, exercise, and stress, play a role in the pathophysiological mechanisms of migraine, affecting the frequency and intensity of symptoms and determining a higher prevalence of psychiatric comorbidity with anxiety and depression as well [9] (Table 2).

Several sleep disorders, including insomnia, obstructive sleep apnea syndrome and restless legs syndrome are associated with migraine. This relationship may be bidirectional: sleep disorders may worsen migraine as well as migraine characteristics, such as frequency, intensity, timing, and daily disability, may predict sleep disturbance in children and adolescents [10].

Literature suggests that a specific dietary style, able to prevent overweight and obesity, is associated with a successful progression of migraine [11].

In addition, inadequate physical activity appears to be associated with an increased risk of migraine (50%) in adolescents [12].

The relationship between exercise and migraine can be complicated; regular exercise has positive effects on chronic migraine, but at the same time it can often trigger migraine attacks, causing avoidance of physical activity itself in some cases [13,14].

Due to the potential impact of body weight on migraine, interventions aimed to modify BMI (body mass index) may be relevant in reducing the severity of the condition [4,15].

Psychological assessment and psychotherapy can prevent a negative cycle between weight, anxiety, and migraine [8].

Stress management is a key coping strategy for any recurring medical condition, therefore screening for stress and mood disorders is essential, in order to refer patients to necessary treatments. Stress has been described as a promoter of the onset of migraine and its chronification, moreover a higher migraine frequency has been associated with higher levels of perceived stress [16,17].

Excessive use of electronic devices is also considered to have a negative impact on physical health, helping the development of sleep disorders, psychosocial problems, musculoskeletal problems, headaches, obesity and psychiatric disorders [18,19,20].

Although available data are limited, the potential benefits, the minimal potential harm and the high prevalence of unhealthy habits in children and adolescents with migraine, support patients’ education on healthy lifestyle habits.

#### 3.1.1. Diet and Food Triggers

As aforementioned diet can play a key role in migraine. Glucose metabolism is relevant in this condition [21], indeed the ketogenic diet has shown to provide some benefits in migraine management [22]. However, weight loss in obese individuals, low-calorie diets, or fatty acid supplementation, might be able to improve migraine [11]. Irregular meal consumption has also been associated with migraine in adolescence [23].

Dietary and nutritional aspects in children have been widely investigated with controversial results. Papetti and colleagues recently shed light on these false beliefs [4], highlighting how there is no evidence of a direct correlation between food allergies and headache, nor is there a role of allergic mechanisms (IgE-mediated) in migraine in children, in which often allergic screening and therapy are routinely assessed. However, it appears that oligoantigenic diets may provide some benefit in certain cases, probably by decreasing the presence of food triggers [24] more than acting on allergic mechanisms.

Therefore, specific foods should not be avoided beforehand, but posology, time of use, and predisposing factors should be investigated for specific food triggers [25].

Food triggers (such as chocolate, caffeine, alcohol, nitrites, aspartame, and gluten) may contribute to the onset of migraine episodes in children as reported in adults as well.

Indeed, all these factors implicated in the diet–migraine relationship share inflammation as a common pathophysiologic target, showing an impact on energy homeostasis, neuropeptide release modulation, neuroreceptors, ion channels and nitric oxide release, vasodilation, and vasoconstriction [26,27].

#### 3.1.2. Obesity/Overweight

The previously mentioned aspects are also connected with obesity/overweight frameworks, in which an active pro-inflammatory state is characterized by higher levels of pro-inflammatory cytokines, alterations in the homeostasis of the adipocytokines-leptin system, adiponectin related to hyperinsulinemic states, modification of energy metabolism, and mitochondrial oxidative stress [4].

The prevalence of obesity/overweight in migrainous children and adolescents is around 50–60% [8,12]. In paediatric age, a 40% increased risk of headache in obese/overweight children is reported, [12] showing both a higher rate of migraine attacks and a more severe headache [12,28,29,30].

Moreover, the percentage of overweight or obese children among chronic daily headache paediatric patients may be almost twice as high as the one of normal-weight children (12%), also with a linkage to a higher comorbidity of anxious and depressive symptoms [3].

#### 3.1.3. Ketogenic Diet

Various approaches are being used to develop new therapeutic strategies, including dietary changes, from the classical ketogenic diet (KD) to the low-calorie ketogenic diet (very-low-calorie VLC-KD) or the modified Atkins diet, along with lifestyle changes (physical activity and psychotherapy, also acting on anxiety and depression) [8,31,32].

The KD is a method of feeding that leads to an increase in the production of ketone bodies (β-hydroxybutyrate, acetoacetate and acetone) in the body and, therefore, to a condition of ketosis. This effect is achieved by obtaining the greatest amount of energy from fats and minimising the consumption of carbohydrates.The ketogenic diet in migraine shows significant potential benefits, observed in a growing number of recent clinical studies [33]. In adults, KD can be a useful alternative, particularly for those who require a low-caloric diet to shed weight, as it affects multiple factors that are associated with migraine [34,35,36]. Several experimental models showed that KD is able to inhibit neuroinflammation by decreasing the proinflammatory cytokines and the formation of ketone bodies and also by acting on the prevention of a hyperinsulinemic state and mitochondrial oxidative stress [34,35,36,37,38,39]. It has been demonstrated that KD reduces the propagation of cortical spreading depression [40] by promoting GABAergic transmission [4,37]. In childhood, few case reports have been reported, in which a KD or VLC-KD has been administered with mixed results. Particularly, the case report by Gburek-Augustat et al. demonstrates that a six-year-old patient suffered from migraines, approximately 6 episodes a year, without triggering factors. The course was characterised by blurred vision followed by hemiparesis. The affected side of the body changed between one attack and another. Hemiplegic migraine appears to be a rare and poorly understood symptom of Glut1 deficiency syndrome, and KD may have positive effects on symptom resolution and improvement of related symptoms [41]. The side effects of the various KDs are reported in a large percentage of cases and are often the cause of poor adherence to the diet [42], the most common being gastrointestinal, cardiovascular, renal/genitourinary and skeletal [43].

#### 3.1.4. Nutraceuticals

The role of nutraceuticals is becoming very important in the prophylactic treatment of migraine in children. Nutraceuticals have the advantage of being very tolerable molecules, as they are also commonly present in daily nutrition [44]. As regards doses and modes of administration of some of these molecules, studies are controversial, and more evidence is needed to establish a unanimous shared range. For other medications, such as magnesium, riboflavin, and melatonin, there are recommended dosages, as shown below. Moreover, although not totally free from adverse effects, these molecules also show a low risk of side effects when compared to traditional drug therapies [45,46]. Magnesium (MG), riboflavin, melatonin, vitamin D, coenzyme Q10, and polyunsaturated fatty acids are reported to be the most commonly applied nutraceuticals for migraine treatment also in paediatrics [4].

These substances appear to take part in the regulation of various vascular and neuronal mechanisms, including the neuroinflammatory block, mitochondrial oxidative stress (MG; melatonin, riboflavin), block of calcium channels, the activity of serotonin receptors (MG; VIT D), effects on CGRP synthesis (MG; melatonin), and N-methyl-D-aspartate glutamate receptor block (MG) [47,48,49,50,51].

Reduced magnesium levels could also contribute to a state of central neuronal hyperexcitability, which is one of the investigated pathophysiological mechanisms of migraine [52]. In paediatric patients with migraine, intracellular magnesium deficiency has been described; up to 9 mg/kg/day supplementation therapy appears to be well tolerated in adolescents. But at present, while several studies reported an effective result in the use of intravenous magnesium in the acute migraine attack, there are still no randomized studies in the literature regarding paediatric patients [48].

About the use of riboflavin, a prophylactic use of 50–400 mg/day is recommended, even in school-age children, providing a reduction in the frequency and duration of attacks. However, retrospective studies in children and adolescents are not as valid as in adults [53]. Recent studies have shown that melatonin can play a role in both prophylaxis and the acute treatment of migraine. For prevention, a dose of 0.3 mg/kg/day is recommended, with effectiveness in around 62% of cases. For acute treatment, a higher efficacy was described with a dosage of 4–8 mg compared to 1–2 mg, although clinical data are still limited [54].

The recent randomized trial by Fallah et al. [55] showed that the combination of topiramate and vitamin D3 was more effective than topiramate alone in reducing the monthly frequency of headache episodes and consequently the impairment of daily functioning in migrainous children. Further research on vitamin D3 treatment would be helpful to verify its clinical efficacy in paediatric migraine prophylaxis.

#### 3.1.5. Palmitoylethanolamide

New trial studies, including the research of Papetti and colleagues [56], assessed the use of palmitoylethanolamide (PEA), an endogenous fatty acid amide largely distributed in several tissues including nervous tissue, also in paediatric migraine treatment.

PEA has significant neuroprotective actions by anti-inflammatory actions and the modulation of mast cell activation and degranulation even at the central level, by analgesic action, showing efficacy in animal models of chronic pain and inflammation, reported in many clinical studies of different painful and inflammatory conditions [57,58].

The application of the neuroprotective and analgesic actions of PEA is emerging as a possible therapeutic strategy also in various paediatric conditions, encompassing both neurodevelopmental disorders (e.g., autism) and the prophylactic treatment of paediatric migraine as well [56,59,60]. Ultramicronized PEA (umPEA) was administered orally 600 mg/day to patients with an average age of 10.3 ± 2.7 and a diagnosis of migraine without aura (ICHD 3 criteria), as part of Papetti and colleagues’ research. They also compared the frequency and intensity of attacks after 3 months of therapy; headache frequency was reduced by >50% per month in more than 60% of patients, and the percentage of patients with severe attacks significatively decreased after treatment (from 8.2% to 1.6%) [56].

### 3.2. Non-Invasive Neuromodulation

Although several studies reported the efficacy of these treatments in both prophylaxis and the management of the acute migraine attacks in adults, there is sparse evidence to support their use in children and young people [61,62]. Based mainly on case reports and case series, we will briefly go over their potential application in the paediatric age and related clinical evidence (Table 3).

#### 3.2.1. Transcutaneous Supraorbital Electrostimulation and External Trigeminal Nerve Stimulator (e-TNS)

The first device approved by the Food and Drug Administration (FDA) for the preventive management of episodic migraine was Cefaly^®^. In children, it has been approved above the age of 8 years (in the US) [63]. It is a novel non-invasive supraorbital transcutaneous device stimulating the supratrochlear and supraorbital branches of the ophthalmic nerve [64].

Numerous studies have shown its effectiveness in the management of migraine, both acute and chronic, giving a good response in 65% of cases [64,65].

#### 3.2.2. Non-Invasive Vagal Nerve Stimulator

The usefulness and benefits of non-invasive vagal nerve stimulator (nVNS) are supported by several studies, especially for the acute treatment of migraine and cluster headaches [66,67].

The PRESTO study showed the efficacy of the Gammacore^®^ nVNS device in reducing migraine attacks in patients with catamenial migraine [68] and a pilot study (EVENT study) showed encouraging results in the prophylaxis of chronic migraine [69].

#### 3.2.3. Transcranial Magnetic Stimulator

The single-pulse transcranial magnetic stimulator (sTMS) has been approved for adult prevention and treatment of chronic migraine and episodic migraine, with a positive impact in reducing the frequency and severity of acute migraine with aura and migraine without aura attacks [70,71]. In particular, Clarke and colleagues reported a reduction in pain intensity of 75%, while Lipton and colleagues demonstrated an efficacy over the placebo in pain freedom of 39% vs. 22% [71].

In paediatric neurological and neuropsychiatric conditions, the repetitive pulse transcranial magnetic stimulator (rTMS) has found application particularly in adolescent depression, autism, and tics/Tourette’s syndrome, due to the dysfunction between the limbic system and the dorsolateral prefrontal cortex (DLPF) found in these neuropsychiatric disorders [72].

However, rTMS has not been sufficiently studied for migraine in children; indeed, protocol adjustments in timing and intensity of released pulses may be necessary because of age-related differences in cranial thickness, trait length, connectivity, and myelination.

#### 3.2.4. Remote Electrical Neuromodulation

The remote electrical neuromodulation (REN) device (Nerivio^®^, Theranica BioElectronics Ltd., Netanya, Israel) is a wireless, wearable, battery-operated stimulation unit, and it is controlled by a smartphone software application, useful for the acute treatment of migraine attacks.

The device generates a signal in the arm peripheral pain sensorial fibres which is thought to modulate pain sensation by the activation of descending inhibitory pain pathways.

Hershey and colleagues performed an open-label, single-arm, multicentre study in migrainous adolescents aged 12–17 years; from this study, 71% of patients obtained pain relief, 35% of participants were pain-free at 2 h, and 69% of participants showed functional ability improvement [73].

### 3.3. Mind–Body Therapies

The failure of migraine symptomatic treatment and prophylaxis may be the consequence of underlying undiagnosed or untreated psychopathological conditions; strong evidence of an association between depressive disorder, anxiety symptoms, and migraine has indeed been highlighted, maybe due to common etiological factors such as the dysfunction of a “neuro-limbic” pain network.

Therefore, psychotherapy and bio-behavioural treatments have been shown to play a key role in the management of migraine and primary headache (Table 4).

#### 3.3.1. Biofeedback

Biofeedback (BFB) is defined by The Association for Applied Psychophysiology and Biofeedback (AAPB) as “a process that enables an individual to learn how to change physiological activity for the purposes of improving health and performance. Precise instruments measure physiological activity such as brainwaves, heart function, breathing, muscle activity, and skin temperature. These instruments rapidly and accurately “feedback” information to the user. The presentation of this information—often in conjunction with changes in thinking, emotions, and behaviour—supports desired physiological changes. Over time, these changes can endure without continued use of an instrument” [74].

Biofeedback often finds implication in pain management, improving self-regulation and the development of coping strategies [75].

Several reviews recommend BFB as successful treatment for migraine and tension headache, showing significant symptom reduction persisting also over long-term follow up [75,76].

Studies conducted on children and adults showed a significant decrease in headache attack frequency after BFB treatment, with an important reduction in preventive drugs’ use [77,78]; moreover, Rausa et al. [79] reported great results of BFB treatment in medication overuse headache, describing a reduction in attack frequency and medication assumption.

#### 3.3.2. Cognitive Behavioural Therapy

Considering migraine in a biopsychosocial context as influenced by biological, cognitive, emotional, and environmental factors, psychological treatment, including cognitive behavioural therapy (CBT), should be considered.

CBT is based on cognitive and behavioural approaches useful for the patient’s management of environmental factors and emotions, thought determination, suppositions, and event interpretation acting as stressor events, therefore worsening or maintaining headaches [80].

Six CBT components have been identified by Noel et al. [81].

- Psychoeducation: the whole family needs to be made aware about headache features together with therapeutic and prophylactic strategies to better deal with the condition.
- Self-monitoring of precipitating factors to provide trigger identification.
- Coping strategies for children with the aim of better coping with pain, such as diaphragmatic breathing and gradual muscle relaxation.
- Parent training in view of providing behavioural strategies to better manage the child’s pain.
- Relapse prevention by offering problem-solving and coping strategies to the whole family.
- Homework (action plan): the whole family needs to be given homework concerning strategies acquired during treatment, so that the patient is given the possibility to apply the discussed and learned competencies also to daily life.

Significant effects on attack frequency decrease have been reported in addition to disability and life quality improvement, together with depression and anxiety symptoms’ reduction [82].

CBT has in fact been described to show better effects in patients with concomitant environmental triggers such as work-related stress, mood disorders, or adapting problems.

In particular, Andrasik et al. (2018) reported that migraine improvement might be correlated with the downgrading of concomitant symptoms such as anxiety and depression; it has in fact been observed that the maintenance of a migraine itself might be due to both the dysfunction of multiple brain areas or to behavioural reactions to stimuli including pain or stress [83].

Ng, Qin Xiang et al. [84] conducted a systematic review and meta-analysis regarding the efficacy of CBT in the management of migraine in children. Results shown a significative and stable improvement in migrainous symptoms in patients undergoing CBT compared to controls, placebo, and standard drugs, both on primary and secondary endpoints on episodic migraine chronic migraine, and chronic daily headache. Migrainous patients’ brain results to have an enhanced reaction to prolonged repeated stimuli, and alteration of inter-ictal information processing is related to limbic system impairment [85].

Brain areas taking part in cognitive and affective pain components are shown to be implicated the same in cognitive interventions, including the prefrontal cortex, midcingulate cortex, thalamus, and amygdala [86].

A study by Powers et al. [87] on migrainous patients investigated CBT benefits when combined with amitriptyline (A) compared to its association with headache education (HE); this study showed that better results were obtained with the combination of CBT plus pharmacological intervention, sustained for at least one year in 86–88% of cases, both on primary and secondary endpoints. Moreover, a similar study by Kroner et al. [88] compared headache frequencies in patients treated with CBT plus A and by HE plus A; this study reported both faster headache frequency reduction and greater stability over time.

#### 3.3.3. Mindfulness

Mindfulness is a therapeutic approach focusing on the present, on body sensations, thought, and emotions including pain, through the encouragement of a perspective of openness, interest, and acknowledgment improving the decisional process and self-efficacy [89].

Great results have been reported in the management of primary headache and chronic pain [89].

The possible effects of mindfulness on biological markers have been hypothesized. A reduction in the levels of pro-inflammatory cytokines and interleukin 6 have been reported. Long-term practice also showed augmentation in telomerase activity and the downregulation of inflammatory response genes [90,91]. Moreover, neuroimaging studies showed variations in the anatomical structure of several brain areas, especially those implicated in affective and cognitive pain components; specifically, augmentation in the activity of the anterior cingulate cortex and anterior insula, activation of the orbito-frontal cortex and deactivation of the thalamus, thickening of cortical areas involved in pain processing, decreased activity in the prefrontal cortex emotional/evaluative areas together with the amygdala and hippocampus, and augmentation in the activity of the mid-cingulate cortex, insula, and thalamus have been reported [92].

## 4. Discussion

The aim of this work was to summarize an up-to-date review of the available literature on the use of non-pharmacological approaches in paediatric migraine. Nutraceutical, non-invasive neuromodulation and behavioural approaches are considered, representing emerging and interesting frontiers in the treatment of migraine also in paediatrics. Unfortunately, even though there are encouraging results in these fields of research, control and randomized studies are still lacking for the youngest migrainous population, without the systematic approaches of investigation.

The nutraceuticals approach is widely spread and well tolerated in paediatrics for the prophylactic treatment of migraine, but again, large population and control studies are missing without univocal guidelines on daily clinical application.

As previously reported, patient comorbidities should be considered in the treatment approaches, with personalized therapy considering the dual efficacy of some therapies in the treatment of both conditions, with bimodal benefit [92]. For instance, using nutraceuticals in children affected by migraine and sleep disorders, for example with vitamin D supplementation and/or melatonin, can help decrease pain related to these conditions [4].

Concerning neuromodulation, on one hand, numerous advantages have been highlighted mostly in adults, linked with the ease of application, infrequency of adverse effects, and high tolerability of these procedures. On the other hand, systematic studies have not reached a wide paediatric population considering the physical and developmental limits in the youngest, for example, concerning age-related differences in cranial thickness for the timing and intensity of released pulses in TMS, as well as the trait length, connectivity, and myelination [72]. Again, future trials are first needed to better confirm the interesting results also in children and adolescents.

Finally, behavioural approaches reached a safe and well-tolerated application in paediatrics, with an efficacy related to both primary and secondary endpoints. Moreover, a combined approach with pharmacological treatments presented a more significant intervention, as reported in several paediatric studies [87,88].

Indeed, behavioural therapies presented encouraging results in reducing headache frequency and severity by mostly acting on the frequent comorbidities represented by depressive and anxious symptoms and disorders [4,9]. These results seem to be stable during the follow up with ameliorative actions on the headache prognosis. Several biomarkers and neuroimaging studies supported this evidence in adults, but more studies are needed in children and adolescents [92].

This narrative review presented an up-to-date review of the current literature in paediatric migraine treatment, considering the three main non-pharmacological approaches and their feedback related to primary and secondary endpoints. Thus, the strength of this work was to draw a more complete landscape on all the supportive or alternative interventions for the treatment of acute and chronic migraine in children and adolescents. As a limit, we underlined the non-systematic nature of our work which may have resulted in not evaluating certain studies. Moreover, research often focuses on clinical trials that do not yet have long-term results. The latter could contribute to the implementation of more reliable protocols and applicable to the entire paediatric and adolescent population.

## 5. Conclusions

Migraine is a complex and disabling condition. The origin is multifactorial, with a genetic predisposition interacting with biological and environmental factors determining its course. Recent decades have seen a change in the conception of the disease, with the application of the biopsychosocial model, as established by the WHO (World Health Organization) through the ICF (International Classification of Functioning, Disability and Health). Therefore, it is essential to define the condition of individuals with migraine as an expression of the interaction of the biological, psychological, and social factors involved, which show a high impact on the development and chronicization of the disease. The effectiveness of non-pharmacological approaches, including nutraceuticals, behavioural approaches, lifestyle modification, and non-invasive neuromodulation, received great interest in the recent literature for treating primary headaches. Thus, an integrated intervention seems to be the most appropriate in the management of these conditions. Considering data collected in this literature review, there was limited evidence of randomized controlled trials regarding non-pharmacological treatments of paediatric migraine. Moreover, among the non-pharmacological strategies in youngsters, a significant effectiveness has been reported only for cognitive behavioural therapy, biofeedback, and relaxation therapy.

It is important to organize a personalized therapy for the paediatric age, since children report different necessities compared to adults. In fact, there are also important limitations in both acute and prophylactic pharmacological treatment. The lack of large, randomized studies in this age group represents one of the main limits, together with the significant placebo effect reported in paediatrics, higher than in adults, with an efficacy in up to 50% of cases, almost equal to the drug therapy efficacy results.

Therefore, more studies are needed evaluating the long-term effects and efficacy of these different approaches, including more standardized protocols that can be generalized to larger patient populations.

## Figures and Tables

**Table 1:** Non Pharmacological therapeutic approaches in paediatric age.

Lifestyle & Nutraceuticals	Non-Invasive Neuromodulation	Mind-Body Therapies
Food Triggers Obesity/Overweight Ketogenic diet Magnesium Riboflavin Melatonin Vitamin D PEA (Palmitoylethanolamide)	eTNS (Transcutaneous supraorbital electrostimulation and external trigeminal nerve stimulator) nVNS (Non-invasive vagal nerve stimulator) TMS (Transcranial magnetic stimulator) REN (Remote electrical neuromodulation)	BFB (Biofeedback treatment) CBT (Cognitive behavioral therapy) MDT (Mindfulness)

**Table 2:** Role and potential application of behavioural approach and lifestyle modifications in migraine in paediatric age.

	Food Triggers (Chocolate)	Food Triggers (Chocolate)	Food Triggers (Chocolate)	Food Triggers (Chocolate)	Food Triggers (Chocolate)
Efficacy or effect in migraine in children	Contribute to the onset of migraine episodes	Predisposing factor	Effective in reducing hemiplegic migraine	Play a role in prophylactic and acute attack treatment of migraine	Possible therapeutic strategy in neuroinflammatory conditions, neurodevelopmental disorders and in paediatric migraine
Mechanism of action	Neuropeptide release modulation; neuroreceptors, ion channels and nitric oxide release; vasodilation and vasoconstriction.	Pro-inflammatory state induction; alterations in the homeostasis of adipocytokines-leptin system; modification of energy metabolism and mitochondrial oxidative stress.	Neuroinflammation inhibition; propagation of cortical spreading depression reduction, promoting GABAergic transmission.	Neuroinflammatory block, mitochondrial oxidative stress, block of calcium channels, activity of serotonin receptors	Anti-inflammatory and analgesic action

**Table 3:** Non-invasive neuromodulation approaches and their potential application in migraine in paediatric age.

Device	e-TNS	n-VNS	s-TMS	REN
Name	Cefaly^®^	gammaCore^®^	s-TMS mini^®^	Nerivio^®^
Mechanism of action	Trigeminal nerve stimulation	Glutamate suppression in trigeminal nucleus	Threshold of occipital cortex increase	Descending inhibitory pain pathways activation
Efficacy in the management of migraine	Good response in 65% of cases, both in acute and chronic migraine	Reduction of migraine attacks in patients with catamenial migraine; good response in the prophylaxis of chronic migraine	Reduction of pain intensity of 75%; efficacy over the placebo in pain freedom of 39% vs. 22%	Pain relief and functional ability improvement

**Table 4:** Mind-body therapies and their potential application in migraine in paediatric age.

	Biofeedback	Cognitive Behavioural Therapy	Mindfullness
Mechanism of action	Pain management, improving self-regulation and the development of coping strategies	Dysfunction of multiple brain areas (prefrontal cortex, midcingulate cortex, thalamus, amygdala) or enhanced behavioural reactions to stimuli including pain or stress.	Reduction of pro-inflammatory cytokines and interleukin, augmentation in telomerase activity and downregulation of inflammatory response genes
Efficacy in the management of migraine	Significant decrease in headache attacks frequency and medications assumption	Attacks frequency decrease; disability and life quality improvement; depression and anxiety symptoms reduction	Management of primary headache and chronic pain

## Data Availability

The data presented in this study are available on request from the corresponding author (accurately indicate status).

## References

[1] Rosignoli C., Ornello R., Onofri A., Caponnetto V., Grazzi L., Raggi A., Leonardi M., Sascco S. (2022). Applying a biopsychosocial model to migraine: Rationale and clinical implications. J. Headache Pain.

[2] Powers S.W., Hershey A.D., Coffey C.S., Chamberlin L.A., Ecklund D.J., Sullivan S.M., Klingner E.A., Yankey J.W., Kashikar-Zuck S., Korbee L.L. (2016). The Childhood and Adolescent Migraine Prevention (CHAMP) Study: A Report on Baseline Characteristics of Participants. Headache J. Head Face Pain.

[3] Sierpina V., Astin J., Giordano J. (2007). Mind-body therapies for headache. Am. Fam. Physician.

[4] Papetti L., Moavero R., Ferilli M.A.N., Sforza G., Tarantino S., Ursitti F., Ruscitto C., Vigevano F., Valeriani M. (2021). Truths and Myths in Pediatric Migraine and Nutrition. Nutrients.

[5] Öztop D.B., Taşdelen B., PoyrazoğLu H.G., Ozsoy S., Yilmaz R., Şahın N., Per H., Bozkurt S. (2016). Assessment of Psychopathology and Quality of Life in Children and Adolescents with Migraine. J. Child Neurol..

[6] Abu Bakar N., Tanprawate S., Lambru G., Torkamani M., Jahanshahi M., Matharu M. (2015). Quality of life in primary headache disorders: A review. Cephalalgia.

[7] Malone C.D., Bhowmick A., Wachholtz A.B. (2015). Migraine: Treatments, comorbidities, and quality of life, in the USA. J. Pain Res..

[8] Tarantino S., Papetti L., Di Stefano A., Messina V., Ursitti F., Ferilli M.A.N., Sforza G., Moavero R., Vigevano F., Gentile S. (2020). Anxiety, Depression, and Body Weight in Children and Adolescents with Migraine. Front. Psychol..

[9] Oskoui M., Pringsheim T., Billinghurst L., Potrebic S., Gersz E.M., Gloss D., Holler-Managan Y., Leininger E., Licking N., Hershey A.D. (2019). Practice guideline update summary: Pharmacologic treatment for pediatric migraine prevention: Report of the Guideline Development, Dissemination, and Implementation Subcommittee of the American Academy of Neurology and the American Headache Society. Neurology.

[10] Pavkovic I.M., Kothare S.V. (2020). Migraine and Sleep in Children: A Bidirectional Relationship. Pediatr. Neurol..

[11] Soodeh R.J., Zeinab G., Paolo M., Lampl C., Mansoureh T. (2019). Association of diet and headache. J. Headache Pain.

[12] Robberstad L., Dyb G., Hagen K., Stovner L.J., Holmen T.L., Zwart J.-A. (2010). An unfavorable lifestyle and recurrent headaches among adolescents: The HUNT study. Neurology.

[13] Amin F.M., Aristeidou S., Baraldi C., Czapinska-Ciepiela E.K., Ariadni D.D., Di Lenola D., Fenech C., Kampouris K., Karagiorgis G., Braschinsky M. (2018). The association between migraine and physical exercise. J. Headache Pain.

[14] Nadelson C. (2006). Sport and Exercise-induced Migraines. Optom. Vis. Sci..

[15] Bond D.S., Thomas J.G., Lipton R.B., Roth J., Pavlovic J.M., Rathier L., O’Leary K.C., Evans E.W., Wing R.R. (2017). Behavioral Weight Loss Intervention for Migraine: A Randomized Controlled Trial. Obesity.

[16] Kelman L. (2007). The Triggers or Precipitants of the Acute Migraine Attack. Cephalalgia.

[17] Nillofr A., Ahumada S.M., Mostoufi S.M., Wright L.J. (2014). Psychological trauma and functional somatic syndromes: A systematic review and meta-analysis. Psychosom. Med..

[18] Thomée S., Harenstam A., Hagberg M. (2011). Mobile phone use and stress, sleep disturbances, and symptoms of depression among young adults-a prospective cohort study. BMC Public Health.

[19] Punamäki R., Wallenius M., Nygård C., Saarni L., Rimpelä A. (2007). Use of information and communication technology (ICT) and perceived health in adolescence: The role of sleeping habits and waking-time tiredness. J. Adolesc..

[20] Cerutti R., Presaghi F., Spensieri V., Valastro C., Guidetti V. (2016). The potential impact of internet and mobile use on headache and other somatic symptoms in adolescence. A population-based cross-sectional study. Headache.

[21] Del Moro L., Rota E., Pirovano E., Rainero I. (2022). Migraine, brain glucose metabolism and the ‘neuroenergetic’ hypothesis: A scoping review. J. Pain.

[22] Di Lorenzo C., Ballerini G., Barbanti P., Bernardini A., D’Arrigo G., Egeo G., Frediani F., Garbo R., Pierangeli G., Prudenzano M.P. (2021). Applications of Ketogenic Diets in Patients with Headache: Clinical Recommendations. Nutrients.

[23] Moschiano F., Messina P., D’amico D., Grazzi L., Frediani F., Casucci G., D’onofrio F., Demurtas A., Beghi E., Bussone G. (2012). Headache, eating and sleeping behaviors and lifestyle factors in preadolescents and adolescents: Preliminary results from an Italian population study. Neurol. Sci..

[24] Egger J., Wilson J., Carter C., Turner M., Soothill J. (1983). IS MIGRAINE FOOD ALLERGY?: A Double-blind Controlled Trial of Oligoantigenic Diet Treatment. Lancet.

[25] Taheri S. (2017). Effect of exclusion of frequently consumed dietary triggers in a cohort of children with chronic primary headache. Nutr. Heal..

[26] Levi-Montalcini R., Skaper S.D., Dal Toso R., Petrelli L., Leon A. (1996). Nerve growth factor: From neurotrophin to neurokine. Trends Neurosci..

[27] Aloe L., Leon A., Levi-Montalcini R. (1993). A proposed autacoid mechanism controlling mastocyte behaviour. Agents Actions.

[28] Ravid S. (2014). Migraine & paediatric obesity: A plausible link?. Indian J. Med. Res..

[29] Hershey A.D., Powers S.W., Nelson T.D., Kabbouche M.A., Winner P., Yonker M., Linder S.L., Bicknese A., Sowel M.K., McClintock W. (2009). Obesity in the pediatric headache population: A multicenter study. Headache J. Head Face Pain.

[30] Kinik S., Alehan F., Erol I., Kanra A. (2009). Obesity and Paediatric Migraine. Cephalalgia.

[31] Verrotti A., Di Fonzo A., Agostinelli S., Coppola G., Margiotta M., Parisi P. (2012). Obese children suffer more often from migraine. Acta Paediatr..

[32] Chai N.C., Bond D.S., Moghekar A., Scher A.I., Peterlin B.L. (2014). Obesity and Headache: Part II—Potential Mechanism and Treatment Considerations. Headache J. Head Face Pain.

[33] Dyńka D., Kowalcze K., Paziewska A. (2022). The Role of Ketogenic Diet in the Treatment of Neurological Diseases. Nutrients.

[34] Kim D.Y., Davis L.M., Sullivan P.G., Maalouf M., Simeone T.A., van Brederode J., Rho J.M. (2007). Ketone bodies are protective against oxidative stress in neocortical neurons. J. Neurochem..

[35] Maalouf M., Sullivan P.G., Davis L., Kim D.Y., Rho J.M. (2007). Ketones inhibit mitochondrial production of reactive oxygen species production following glutamate excitotoxicity by increasing NADH oxidation. Neuroscience.

[36] Jeong E.A., Jeon B.T., Shin H.J., Kim N., Lee D.H., Kim H.J., Kang S.S., Cho G.J., Choi W.S., Roh G.S. (2011). Ketogenic diet-induced peroxisome proliferator-activated receptor-γ activation decreases neuroinflammation in the mouse hippocampus after kainic acid-induced seizures. Exp. Neurol..

[37] Yudkoff M., Daikhin Y., Horyn O., Nissim I., Nissim I. (2008). Ketosis and brain handling of glutamate, glutamine, and GABA. Epilepsia.

[38] Waeber C., Moskowitz M.A. (2005). Migraine as an inflammatory disorder. Neurology.

[39] Borkum J.M. (2016). Migraine triggers and oxidative stress: A narrative review and synthesis. Headache J. Head Face Pain.

[40] De Almeida Rabello Oliveira M., da Rocha Ataíde T., de Oliveira S.L., de Melo Lucena A.L., de Lira C.E.P.R., Soares A.A., de Almeida C.B.S., Ximenes-da-Silva A. (2008). Effects of short-term and long-term treatment with medium- and long-chain triglycerides ketogenic diet on cortical spreading depression in young rats. Neurosci. Lett..

[41] Gburek-Augustat J., Heinze A., Jamra R.A., Merkenschlager A. (2020). Hemiplegic Migraine in Glut1 Deficiency Syndrome and Paroxysmal Dyskinesia at Ketogenic Diet Induction: Case Report and Literature Review. Mov. Disord. Clin. Pract..

[42] Martin-McGill K.J., Bresnahan R., Levy R.G., Cooper P.N. (2020). Ketogenic diets for drug-resistant epilepsy. Cochrane Database Syst. Rev..

[43] Cai Q.-Y., Zhou Z.-J., Luo R., Gan J., Li S.-P., Mu D.Z., Wan C.-M. (2017). Safety and tolerability of the ketogenic diet used for the treatment of refractory childhood epilepsy: A systematic review of published prospective studies. World J. Pediatr..

[44] Orr S.L. (2018). The Evidence for the Role of Nutraceuticals in the Management of Pediatric Migraine: A Review. Curr. Pain Headache Rep..

[45] Rajapakse T., Pringsheim T. (2016). Nutraceuticals in Migraine: A Summary of Existing Guidelines for Use. Headache J. Head Face Pain.

[46] Prieto J.M. (2014). Update on the efficacy and safety of Petadolex®, a butterbur extract for migraine prophylaxis. Botanics.

[47] Werner K., Qaiser S., Kabbouche M., Murphy B., Maconochie I., Hershey A.D. (2020). Intravenous migraine treatment in children and adolescents. Curr. Pain. Headache Rep..

[48] Maier J.A., Pickering G., Giacomoni E., Cazzaniga A., Pellegrino P. (2020). Headaches and Magnesium: Mechanisms, Bioavailability, Therapeutic Efficacy and Potential Advantage of Magnesium Pidolate. Nutrients.

[49] Gelfand A.A., Ross A.C., Irwin S.L., Greene K.A., Qubty W.F., Allen I.E. (2020). Melatonin for acute treatment of migraine in children and adolescents: A pilot randomized trial. Headache J. Head Face Pain.

[50] Fallah R., Shoroki F.F., Ferdosian F. (2015). Safety and Efficacy of Melatonin in Pediatric Migraine Prophylaxis. Curr. Drug Saf..

[51] Fallah R., Fazelishoroki F., Sekhavat L. (2018). A Randomized Clinical Trial Comparing the Efficacy of Melatonin and Amitriptyline in Migraine Prophylaxis of Children. Iran. J. Child Neurol..

[52] Castelli S., Meossi C., Domenici R., Fontana F., Stefani G. (1993). Magnesium in the prophylaxis of primary headache and other periodic disorders in children. Pediatr. Medica Chir. Med. Surg. Pediatr..

[53] Yamanaka G., Suzuki S., Takeshita M., Go S., Morishita N., Takamatsu T., Daida A., Morichi S., Ishida Y., Oana S. (2020). Effectiveness of low-dose riboflavin as a prophylactic agent in pediatric migraine. Brain Dev..

[54] Zduńska A., Cegielska J., Domitrz I. (2022). The Pathogenetic Role of Melatonin in Migraine and Its Theoretic Implications for Pharmacotherapy: A Brief Overview of the Research. Nutrients.

[55] Fallah R., Yazd S.S., Sohrevardi S.M. (2020). Efficacy of topiramate alone and topiramate plus vitamin D3 in the prophylaxis of pediatric migraine: A randomized clinical trial. Iran. J. Child Neurol..

[56] Papetti L., Sforza G., Tullo G., di Loro P.A., Moavero R., Ursitti F., Ferilli M.A.N., Tarantino S., Vigevano F., Valeriani M. (2020). Tolerability of Palmitoylethanolamide in a Pediatric Population Suffering from Migraine: A Pilot Study. Pain Res. Manag..

[57] Petrosino S., Di Marzo V. (2016). The pharmacology of palmitoylethanolamide and first data on the therapeutic efficacy of some of its new formulations. Br. J. Pharmacol..

[58] Artukoglu B.B., Beyer C., Zuloff-Shani A., Brener E., Bloch M.H. (2017). Efficacy of Palmitoylethanolamide for Pain: A Meta-Analysis. Pain Physician.

[59] Chirchiglia D., Cione E., Caroleo M.C., Wang M., Di Mizio G., Faedda N., Giacolini T., Siviglia S., Guidetti V., Gallelli L. (2018). Effects of Add-On Ultramicronized N-Palmitol Ethanol Amide in Patients Suffering of Migraine with Aura: A Pilot Study. Front. Neurol..

[60] Khalaj M., Saghazadeh A., Shirazi E., Shalbafan M.-R., Alavi K., Shooshtari M.H., Laksari F.Y., Hosseini M., Mohammadi M.-R., Akhondzadeh S. (2018). Palmitoylethanolamide as adjunctive therapy for autism: Efficacy and safety results from a randomized controlled trial. J. Psychiatr. Res..

[61] Loh N.R., Whitehouse W.P., Howells R. (2022). What is new in migraine management in children and young people?. Arch. Dis. Child..

[62] Andrasik F., Grazzi L., Sansone E., D’Amico D., Raggi A., Grignani E. (2018). Non-pharmacological approaches for headaches in young age: An updated review. Front. Neurol..

[63] Schoenen J., Roberta B., Magis D., Coppola G. (2016). Noninvasive neurostimulation methods for migraine therapy: The available evidence. Cephalalgia.

[64] Schoenen J.E. (2016). Migraine prevention with a supraorbital transcutaneous stimulator: A randomized controlled trial. Neurology.

[65] Russo A., Tessitore A., Conte F., Marcuccio L., Giordano A., Tedeschi G. (2015). Transcutaneous supraorbital neurostimulation in ‘de novo’ patients with migraine without aura: The first Italian experience. J. Headache Pain.

[66] Yuan H., Silberstein S.D. (2016). Vagus nerve and vagus nerve stimulation, a comprehensive review: Part I. Headache J. Head Face Pain.

[67] Goadsby P., Grosberg B., Mauskop A., Cady R., Simmons K. (2014). Effect of noninvasive vagus nerve stimulation on acute migraine: An open-label pilot study. Cephalalgia.

[68] Russo A., Tessitore A., Conte F., Marcuccio L., Giordano A., Tedeschi G. (2018). Noninvasive vagus nerve stimulation as acute therapy for migraine: The randomized PRESTO study. Neurology.

[69] Silberstein S.D., Calhoun A.H., Lipton R.B., Grosberg B.M., Cady R.K., Dorlas S., Simmons K.A., Mullin C., Liebler E.J., EVENT Study Group (2016). Chronic migraine headache prevention with noninvasive vagus nerve stimulation: The EVENT study. Neurology.

[70] Lipton R.B., Dodick D.W., Silberstein S.D., Saper J.R., Aurora S.K., Pearlman S.H., Fischell R.E., Ruppel P.L., Goadsby P.J. (2010). Single-pulse transcranial magnetic stimulation for acute treatment of migraine with aura: A randomised, double-blind, parallel-group, sham-controlled trial. Lancet Neurol..

[71] Clarke B.M., Upton A.R.M., Kamath M.V., Al-Harbi T., Castellanos C.M. (2006). Transcranial magnetic stimulation for migraine: Clinical effects. J. Headache Pain.

[72] Malone L.A., Sun L.R. (2019). Transcranial Magnetic Stimulation for the Treatment of Pediatric Neurological Disorders. Curr. Treat. Options Neurol..

[73] Hershey A.D., Lin T., Gruper Y., Harris D., Ironi A., Berk T., Szperka C.L., Berenson F. (2020). Remote electrical neuromodulation for acute treatment of migraine in adolescents. Headache J. Head Face Pain.

[74] The Association for Applied Psychophysiology and Biofeedback—AAPB https://aapb.org/index.php.

[75] Sun-Edelstein C., Mauskop A. (2011). Alternative headache treatments: Nutraceuticals, behavioural and physical treatments. Headache J. Head Face Pain.

[76] Nestoriuc Y., Martin A., Rief W., Andrasik F. (2008). Biofeedback Treatment for Headache Disorders: A Comprehensive Efficacy Review. Appl. Psychophysiol. Biofeedback.

[77] A Stokes D., Lappin M.S. (2010). Neurofeedback and biofeedback with 37 migraineurs: A clinical outcome study. Behav. Brain Funct..

[78] Blume H.K., Ba L.N.B., Breuner C.C. (2012). Biofeedback Therapy for Pediatric Headache: Factors Associated with Response. Headache J. Head Face Pain.

[79] Rausa M., Palomba D., Cevoli S., Lazzerini L., Sancisi E., Cortelli P., Pierangeli G. (2016). Biofeedback in the prophylactic treatment of medication overuse headache: A pilot randomized controlled trial. J. Headache Pain.

[80] Faedda N., Natalucci G., Baglioni V., Giannotti F., Cerutti R., Guidetti V. (2019). Behavioral therapies in headache: Focus on mindfulness and cognitive behavioral therapy in children and adolescents. Expert Rev. Neurother..

[81] Noel M., Petter M., Parker J.A., Chambers C.T. (2012). Cognitive behavioral therapy for pediatric chronic pain: The problem, research, and practice. J. Cogn. Psychother..

[82] Raggi A., Giovannetti A.M., Quintas R., D’amico D., Cieza A., Sabariego C., Bickenbach J.E., Leonardi M. (2012). A systematic review of the psychosocial difficulties relevant to patients with migraine. J. Headache Pain.

[83] Andrasik F., Flor H., Turk D.C. (2005). An expanded view of psychological aspects in head pain: The biopsychosocial model. Neurol. Sci..

[84] Ng Q.X., Venkatanarayanan N., Kumar L. (2016). A Systematic Review and Meta-Analysis of the Efficacy of Cognitive Behavioral Therapy for the Management of Pediatric Migraine. Headache J. Head Face Pain.

[85] Coppola G., Di Lorenzo C., Serrao M., Parisi V., Schoenen J., Pierelli F. (2016). Pathophysiological targets for non-pharmacological treatment of migraine. Cephalalgia.

[86] Flor H. (2014). Psychological pain interventions and neurophysiology: Implications for a mechanism-based approach. Am. Psychol..

[87] Powers S.W., Kashikar-Zuck S.M., Allen J.R., LeCates S.L., Slater S.K., Zafar M., Hershey A.D. (2013). Cognitive behavioral therapy plus amitriptyline for chronic migraine in children and adolescents: A randomized clinical trial. JAMA.

[88] Kroner J.W., Hershey A.D., Kashikar-Zuck S.M., LeCates S.L., Allen J.R., Slater S.K., Zafar M., Kabbouche M.A., O’Brien H.L., Shenk C.E. (2016). Cognitive Behavioral Therapy plus Amitriptyline for Children and Adolescents with Chronic Migraine Reduces Headache Days to ≤4 Per Month. Headache J. Head Face Pain.

[89] Sansone E., Raggi A., Grignani E., Leonardi M., D’amico D., Scaratti C., Grazzi L. (2018). Mindfulness meditation for chronic migraine in pediatric population: A pilot study. Neurol. Sci..

[90] Raggi A., Grignani E., Leonardi M., Andrasik F., Sansone E., Grazzi L., D’Amico D. (2018). Behavioral Approaches for Primary Headaches: Recent Advances. Headache J. Head Face Pain.

[91] Andrasik F., Grazzi L., D’Amico D., Sansone E., Leonardi M., Raggi A., Salgado-García F. (2016). Mindfulness and headache: A ‘new’ old treatment, with new findings. Cephalalgia.

[92] Grazzi L. (2022). Mindfulness and other behavioral approaches. Neurol. Sci..

