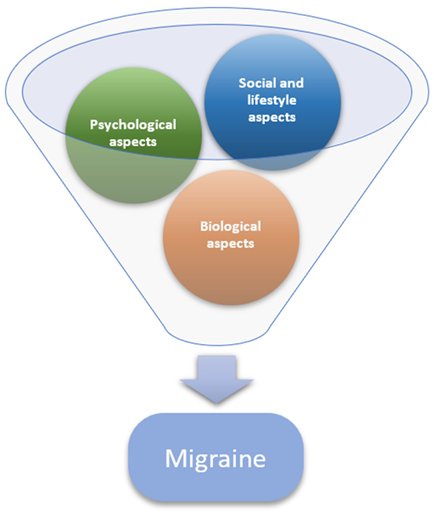# Correction: Baglioni et al. Non-Pharmacological Treatments in Paediatric Migraine. *J. Clin. Med.* 2024, *13*, 1278

**DOI:** 10.3390/jcm15176911

**Published:** 2026-09-07

**Authors:** Valentina Baglioni, Fabiola Bozza, Annachiara Beatrice, Noemi Cameli, Elisa Maria Colacino Cinnante, Giuliana Lentini, Noemi Faedda, Giulia Natalucci, Vincenzo Guidetti

**Affiliations:** Child Neurology and Psychiatry Unit, Department of Human Neuroscience, Sapienza University, Via dei Sabelli 108, 00185 Rome, Italy; fabiola.bozza@uniroma1.it (F.B.); annachiara.beatrice@uniroma1.it (A.B.); noemi.cameli@uniroma1.it (N.C.); elisamaria.colacinocinnante@uniroma1.it (E.M.C.C.); giuliana.lentini@uniroma1.it (G.L.); noemi.faedda@uniroma1.it (N.F.); giulia.natalucci@uniroma1.it (G.N.); vincenzo.guidetti@uniroma1.it (V.G.)

## Error in Figure

In the original publication [1], there was a mistake in Figure 1. Migraine biopsychosocial model, as published. 

Unfortunately, the similarity to Figure 2, published in 2022 in the article “Applying a biopsychosocial model to migraine: rationale and clinical implications” (doi: 10.1186/s10194-022-01471-3), proved to be very evident, although entirely unintentional.

The corrected Figure 1. Migraine biopsychosocial model appears below. The authors state that the scientific conclusions are unaffected. This correction was approved by the Academic Editor. The original publication has also been updated.